# Acetaldehyde Removal from Indoor Air through Chemical Absorption Using L-Cysteine

**DOI:** 10.3390/ijerph7093489

**Published:** 2010-09-17

**Authors:** Kyoko Yamashita, Miyuki Noguchi, Atsushi Mizukoshi, Yukio Yanagisawa

**Affiliations:** 1 Department of Urban Engineering, School of Engineering, The University of Tokyo, Hongo 7-3-1, Bunkyo-ku, Tokyo 113-8656, Japan; 2 Department of Environment Systems, Graduate School of Frontier Sciences, The University of Tokyo, Kashiwa-no-ha 5-1-5, Kashiwa-shi, Chiba 277-8563, Japan E-Mails: miyuki_noguchi@yy.k.u-tokyo.ac.jp (M.N.); yukio@k.u-tokyo.ac.jp (Y.Y.); 3 Tokyo Metropolitan Industrial Technology Research Institute, Nishigaoka 3-13-10, Kita-ku, Tokyo 115-8586, Japan; E-Mail: atsushi_mizukoshi@yy.k.u-tokyo.ac.jp

**Keywords:** acetaldehyde, irreversibly removal, l-cysteine, bubbling method, l-cysteine-containing gel

## Abstract

The irreversible removal of acetaldehyde from indoor air via a chemical reaction with amino acids was investigated. To compare effectiveness, five types of amino acid (glycine, l-lysine, l-methionine, l-cysteine, and l-cystine) were used as the reactants. First, acetaldehyde-laden air was introduced into aqueous solutions of each amino acid and the removal abilities were compared. Among the five amino acids, l-cysteine solution showed much higher removal efficiency, while the other amino acids solutions didn’t show any significant differences from the removal efficiency of water used as a control. Next, as a test of the removal abilities of acetaldehyde by semi-solid l-cysteine, a gel containing l-cysteine solution was put in a fluororesin bag filled with acetaldehyde gas, and the change of acetaldehyde concentration was measured. The l-cysteine-containing gel removed 80% of the acetaldehyde in the air within 24 hours. The removal ability likely depended on the unique reaction whereby acetaldehyde and l-cysteine rapidly produce 2-methylthiazolidine-4-carboxylic acid. These results suggested that the reaction between acetaldehyde and l-cysteine has possibilities for irreversibly removing toxic acetaldehyde from indoor air.

## 1. Introduction

Airborne acetaldehyde is known to have adverse health effects, as exposure to acetaldehyde can induce sensory irritation [1] and acetaldehyde itself is considered a possible human carcinogen [2]. The primary source of acetaldehyde in indoor environments is construction lumber [3]. The secondary emission sources come mainly from combustion of hydrocarbons during cooking [4], smoking [5], and drinking alcohol [6]. Though it is easy to reduce the amount of acetaldehyde emitted from primary sources by avoiding the use of materials in which acetaldehyde is included, reducing the generation from ready-made products and secondary acetaldehyde sources is difficult. Due to the difficulties in reduction of acetaldehyde generation, indoor concentrations haven’t decreased enough in recent years [7].

Current methods to remove acetaldehyde from indoor air include plasma oxidation, photocatalytic oxidation and adsorption by activated carbons. The plasma discharge method is used in domestic air cleaners. The radicals formed by plasma discharge are strong oxidants and degrade acetaldehyde to carbon monoxide and carbon dioxide [8]. However, these radicals also oxidize nitrogen and oxygen simultaneously, generating nitrogen dioxide and ozone, respectively [8,9]. Ozone not only has adverse health effects for humans, but it also reacts with unsaturated volatile organic compounds (VOCs) and generates carbonyl compounds [10], which may also contribute as secondary sources of acetaldehyde in indoor environments.

Photocatalytic oxidation of acetaldehyde is a fairly recent technology. Titania (TiO_2_) and zinc oxide (ZnO) are commonly used as catalysts [11]. Generally a photocatalyst activated by UV radiation generates hydroxyl radicals derived from adsorbed water or hydroxyl ions as the dominant oxidant. The oxidant and atmospheric O_2_ transform VOCs into CO_2_ and H_2_O [11]. Though the photocatalytic purification is recognized as effective for a broad range of VOCs [12], the oxidation reaction sometimes doesn’t proceed to complete degradation, and acetic acid or coke-like materials remain on the surface of the catalyst [13].

Use of activated carbons is a widespread method for reducing airborne acetaldehyde. However, because activated carbon removes VOCs via physical adsorption from the atmosphere, desorption would occur when the ambient concentration is decreased. Moreover, hydrophilic acetaldehyde has low affinity for hydrophobic activated carbon. To overcome these problems, some devices have been investigated. It was reported that an increase in the content of oxygen [14,15] and nitrogen atoms [16] in the activated carbon structure results in an increase of the saturation adsorption limit of acetaldehyde. Coating activated carbon with acetaldehyde-friendly compounds also seems to be effective for increasing removal efficiency. For instance, Hayashi *et al.* studied the use of amine-coated activated carbon for acetaldehyde removal [17]. It is known that compounds with amino groups react with carbonyl compounds giving imines (Schiff bases) and this reaction allows activated carbons coated with amines to remove acetaldehyde more effectively. Though hydrazine compounds which are reactive towards acetaldehyde have been investigated as acetaldehyde adsorbents, they are suspected to be mutagenic [18]. Amino acids, which also contain amino groups in their structures, would also seem to have reactivity with acetaldehyde, but removal of acetaldehyde from indoor air using amino acids has not been studied.

In this study, five amino acids were investigated as reactants because they possess amino groups and are much less toxic than hydrazine compounds. We conducted two kinds of experiments using a bubbling method and a bag method. First, to compare the effectiveness for acetaldehyde removal between amino acids, acetaldehyde-laden air has been introduced into aqueous solutions of each amino acid (bubbling method). Next, to assess the ability to absorb acetaldehyde, acetaldehyde-laden air has been contacted with an l-cysteine-containing gel (bag method), and then the removal efficiency in each experiment has been tested.

## 2. Experimental Section

### 2.1. Bubbling Method

#### 2.1.1. Amino acids

**Figure 1.**
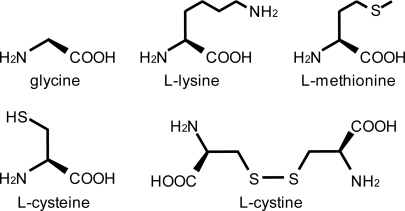
Structures of amino acids used in bubbling method.

Five types of amino acid were investigated in this experiment; glycine, l-lysine, l-methionine, l-cysteine, and l-cystine. Figure 1 shows the structures of these amino acids. Glycine has the simplest structure among amino acids. l-Lysine has two amino groups per molecule. l-Methionine has a methylsulfanyl group in the terminal side chain. l-Cysteine has also a sulfur atom, which exists as a sulfhydryl group. l-Cystine is an oxidized derivative of l-cysteine. These chemicals were all purchased from Wako Chemical.

Each amino acid was dissolved in deionized water (Milli-Q water) and aqueous solutions were thus prepared. The amino group concentration was 3.3–3.4 mM. The exception was l-cystine, which is insoluble in water; thereby an emulsion was prepared to be equivalent to the solutions. The concentrations of aqueous solutions were high enough to react with all the acetaldehyde which was passed through each solution within 1 hour.

#### 2.1.2. Experimental apparatus

**Figure 2.**
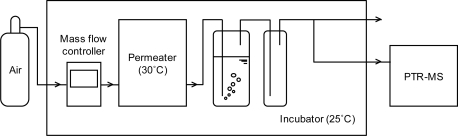
Apparatus for the bubbling method.

Figure 2 shows the bubbling apparatus. Acetaldehyde gas was generated from the permeation tube in a permeater (GASTEC); dry air introduced to the permeater diluted the gaseous acetaldehyde. The flow rate was adjusted to 200 mL min^−1^ by the mass flow controller. As a result, air containing acetaldehyde at concentrations of approximately 1.0 to 1.7 ppm was continuously generated. The acetaldehyde-laden air was introduced into the first jar containing 30 mL of the aqueous solution of an amino acid mentioned above. The second jar was installed to remove water droplets. At the exit of the second jar, the concentration of acetaldehyde was measured using Proton Transfer Reaction Mass Spectrometry (PTR-MS, Ionicon GmbH). The principle and operating condition of PTR-MS will be described below. The inlet concentration was also measured by PTR-MS before and after running the bubbling experiments for five cycles and the average of two measured values was adopted as the inlet concentration of the single trial (each value is shown in Table 1). All tests were carried out three times for each aqueous solution with the exception of the l-lysine and l-methionine solutions, which were investigated once (Table 1).

#### 2.1.3. PTR-MS

PTR-MS is a novel analytical instrument for online measurements of trace amount of VOCs, including oxygenated VOCs such as acetaldehyde [19]. To date, the laboratory based analysis of atmospheric VOCs requires concentrating the trace amount compounds on a particular adsorbent, conducting complicated pretreatment, and operating the analytical instrument. Therefore these methods can be said to be time-consuming. On the other hand, the PTR-MS system has high sensitivity to VOCs and allows direct air inlet and real-time analysis.

In this study, PTR-MS was used to measure gaseous acetaldehyde concentrations. For the bubbling experiments, PTR-MS could track the changes of outlet acetaldehyde concentrations from moment to moment. Analytical conditions used are shown in Table 2.

### 2.2. Bag Method

In this experiment, only l-cysteine, which most reduced the acetaldehyde concentration in the previous bubbling experiments, was used for the reactant. One mL of aqueous l-cysteine solution (4.7 mM) was absorbed into 0.05 g of water-absorbing polymer (cross-linked, acrylic acid/sodium acrylate copolymer; ACRYHOPE^©^, NIPPON SHOKUBAI), thus giving an l-cysteine gel. The gel was settled in a 2 L fluororesin bag and the bag was sealed with a clip. After all of the air was suctioned from inside the bag, acetaldehyde-laden air was introduced from the permeater at a flow rate of 200 mL min^−1^. In total, 1 L of acetaldehyde-laden air was introduced into each bag. The stopcock was closed, then the bag was placed in the incubator and the temperature adjusted at 25 °C for 24 hours. After 24 hours, the acetaldehyde concentration of inside the bag was measured by PTR-MS. The operating conditions of PTR-MS were the same as for the bubbling method experiments (Table 2). For comparison purposes, bags containing water absorbed gel and empty bags were also prepared, so three bags were prepared simultaneously for each condition.

## 3. Results and Discussion

### 3.1. Inlet Concentration

Before the bubbling experiments, the inlet acetaldehyde concentration was measured for five cycles and an average concentration was calculated. In the same way, an average inlet concentration was obtained after the experiment. Coefficients of variance of PTR-MS measurements in five cycles were 0.16%–0.80%, 0.41%–1.3%, 0.78%–3.3%, 0.58%–0.8%, 0.15%–0.94% and 0.42%–1.1% for water, glycine, l-lysine, l-methionine, l-cysteine and l-cystine, respectively. A t-test for comparison of average concentrations of before and after bubbling experiment indicated that there was no significant difference between the two values for all trials except in the case of l-lysine (α = 0.01). The inlet concentration of the single trial (*C_in_*) was then obtained by calculating the average of the two inlet concentration values. Regarding the bag method, inlet concentration was calculated in the same way as in the bubbling method: average concentration before and after introduction the gas into all bags. Again, no significant difference was observed between the two values (α = 0.01).

### 3.2. Bubbling Method

**Figure 3.**
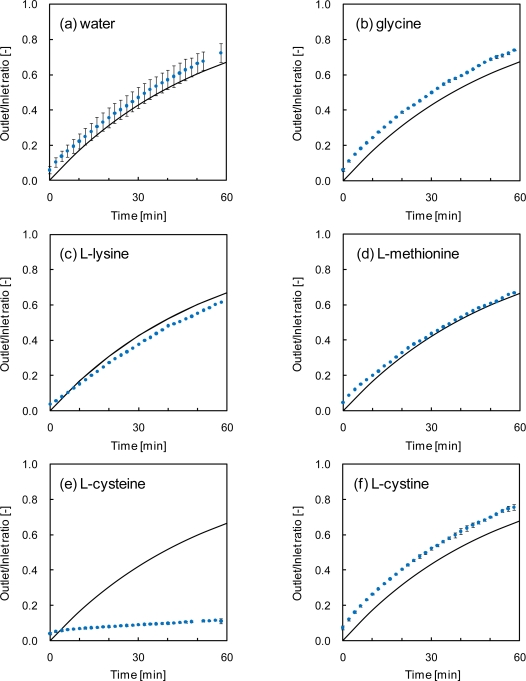
Change in ratio of outlet acetaldehyde concentration to inlet. Solid lines and closed circles correspond to the calculation values for water using Equation (2) and the experimental values, respectively. Error bars represent standard deviation for (a), (b), (e) and (f).

The change in ratio of outlet acetaldehyde concentration to inlet is shown with dots in Figure 3. The outlet acetaldehyde concentration through water and all aqueous amino acid solutions except for l-cysteine increased with elapsed time. If equilibrium was achieved while the gas passed through the solution, the ratio between outlet and inlet partial pressure will be described as in Equation 1:

(1) $$\frac{P_{\text{out}}}{P_{\text{in}}}=1-\text{exp}\left(-\frac{G}{\text{VHRT}}t\right)$$

where *P_in_* is partial pressure of acetaldehyde in inlet gas [Pa], *P_out_* is partial pressure of acetaldehyde in outlet gas [Pa], *G* is gas volumetric flow [m^3^ min^−1^], *V* is fluid volume [m^3^], *H* is Henry constant [mol m^−3^ Pa^−1^], *R* is gas constant [Pa m^3^ K^−1^ mol^−1^], *T* is temperature [K], and *t* is elapsed time [min]. Henry constant used in calculation was 0.15 mol m^−3^ Pa^−1^, for water at 25 °C. Because the acetaldehyde concentration was at trace levels, the ratio of partial pressure could be transformed into a concentration ratio:

(2) $$\frac{C_{\text{out}}}{C_{\text{in}}}=1-\text{exp}\left(-\frac{G}{\text{VHRT}}t\right)$$

where *C_in_* is concentration of acetaldehyde in inlet gas [ppm] and *C_out_* is concentration of acetaldehyde in outlet gas [ppm]. The calculated value using Equation (2) for pure water was drawn with a solid line in Figure 3. The experimental values were in good agreement with the line for Figures 3(a–d) and (f). The absence of a difference between water and the solutions of each amino acid (glycine, l-methionine, l-lysine and l-cystine) indicates that there is no apparent reaction between acetaldehyde and the amino acids due to the hydrolyzability of the corresponding imines.

In contrast, the outlet acetaldehyde concentration through l-cysteine solution didn’t increase with time [Figure 3(e)]. This suggests that acetaldehyde absorbed to the aqueous solution reacted with l-cysteine rapidly, and in sequence the reduction in acetaldehyde concentration in the aqueous phase prompted additional absorption of gas phase acetaldehyde. As a consequence, most of acetaldehyde in the gas phase was removed. The average percentage of acetaldehyde which was removed during a 60-min experimental period was 91% in l-cysteine solution, while they were were 50%–64% in other solutions (Table 3). Therefore the net removal efficiency of l-cysteine solution was 1.5 to 1.7 times higher than that of the other amino acids tested.

**Scheme 1.**
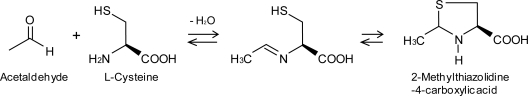
Reaction between acetaldehyde and l-cysteine.

Generally, the reaction between acetaldehyde and an amino acid would generate an imine. The imine generated by reaction between acetaldehyde and hydroxylamine or hydrazine has a delocalized and comparatively stable structure [20], while the reactions between acetaldehyde and glycine, l-lysine, l-methionine or l-cystine only form imines with less stable structures. In the case of acetaldehyde and l-cysteine, however, acetaldehyde rapidly condenses with l-cysteine to give 2-methyl-thiazolidine-4-carboxylic acid (MTCA) through a Schiff base intermediate [21]. This reaction and the structure of MTCA are shown in Scheme 1. The reaction has been studied to protect the inside of the body from damage by acetaldehyde formed by ethanol metabolism in the liver [22]. Rapid production of MTCA would result in a decreasing dissolved acetaldehyde concentration; thereby more gaseous acetaldehyde would be absorbed in the bubbling experiments and therefore, the outlet acetaldehyde concentration was below 10% of the inlet value and an increase in the concentration was not observed. The production of MTCA could not be confirmed by H-NMR and FTIR analysis due to the small amount of MTCA present, compared to the initial l-cysteine and its oxidized derivatives such as l-cystine; since the total amount of l-cysteine initially dissolved in 30 mL of aqueous solution was sufficient to react with the acetaldehyde which passed through during the experimental period (60 min), the amount of MTCA produced should be around 7.8 × 10^−7^ mol.

### 3.3. Bag Method

The acetaldehyde concentration in the air introduced into the bags was 1.57 ppm. The average concentrations after 24 hours were 1.37 ± 0.0062 ppm, 1.42 ± 0.018 ppm, and 0.118 ± 0.0055 ppm in the blank bags, the bags of water gel, and of l-cysteine solution gel, respectively. Therefore the removal efficiency in each bag was calculated to be 13 ± 0.39%, 9.5 ± 1.2%, 92 ± 0.30%, respectively. The hypothesis that the reduction in the concentration by 13% inside of blank bag was due to a loss by adsorption on the wall of bag, supports the notion that the water gel didn’t work for removing the acetaldehyde from the gas phase. Regarding the adsorption on the wall of bag, l-cysteine solution gel contributed approximately 80% to the removal of acetaldehyde.

## 4. Conclusions

In this study, five types of amino acid were investigated for irreversible removal of acetaldehyde from air. The result of introducing acetaldehyde gas into each amino acid solution indicates that net removal efficiency of l-cysteine solution is 1.5 to 1.7 times higher than that of the other amino acids used in this study. An l-cysteine-containing gel also removed acetaldehyde from the air by 80% within 24 hours. The reaction between acetaldehyde and l-cysteine thus has possibilities for becoming an application to irreversibly remove toxic acetaldehyde from indoor air. This study demonstrated the removal of acetaldehyde from air by l-cysteine solution and l-cysteine-containing gel, suggesting an l-cysteine-containing absorbent in the form of a liquid and semi-solid, respectively. Therefore preparation of l-cysteine-containing adsorbents in a dry and solid form should be studied for practical use in indoor environments.

## Figures and Tables

**Table 1.** The number of experimental trials and inlet concentration of acetaldehyde of bubbling method.

**Substance**	***n***	**Inlet concentration[ppm]**
Water	3	1.59–1.67
Glycine	3	1.66–1.67
L-Lysine	1	1.11
L-Methionine	1	1.02
L-Cysteine	3	1.58–1.59
L-Cystine	3	1.66–1.68

*n* = the number of trials.

**Table 2.** Analytical conditions of PTR-MS.

**Operation mode**	Multiple Ion Detecting (MID) mode
**Detected ion**	m/z = 45 (acetaldehyde)
**Reaction rate constant**	3.6 × 10^−9^ cm^3^ molecule^−1^ s^−1^
**Dwell time**	60 s

**Table 3.** Average percentage of acetaldehyde removal by a 60-min bubbling method. Figures represent average **±** SD for water, glycine, l-cysteine, l-cystine (n = 3) and average for l-lysine and l-methionine.

**Sample solution**	**Removal rate [%]**
Water	55 ± 5
Glycine	52 ± 0.2
l-Lysine	64
l-Methionine	58
l-Cysteine	91 ± 0.4
l-Cystine	50 ± 1
